# Factors Affecting Cell Viability during the Enzymatic Dissociation of Human Endocrine Tumor Tissues

**DOI:** 10.3390/biology13090665

**Published:** 2024-08-27

**Authors:** Anastasia Shcherbakova, Marina Utkina, Anna Valyaeva, Nano Pachuashvili, Ekaterina Bondarenko, Liliya Urusova, Sergey Popov, Natalya Mokrysheva

**Affiliations:** 1 Department of General, Molecular and Population Genetics, Endocrinology Research Centre, Dm. Ulyanova St., 11, 117292 Moscow, Russia; nastya.shcherbakova1@gmail.com (A.S.); valyaeva.ann@gmail.com (A.V.); pachuashvili.nano@endocrincentr.ru (N.P.); bondarenko.ekaterina@endocrincentr.ru (E.B.); urusova.liliya@endocrincentr.ru (L.U.); swpopov73@gmail.com (S.P.); mokrisheva.natalia@endocrincentr.ru (N.M.); 2 A.N. Belozersky Institute of Physico-Chemical Biology, Lomonosov Moscow State University, Leninskye Gory, House 1, Building 40, 119992 Moscow, Russia

**Keywords:** enzymatic dissociation, endocrine tumors, morphological features, cell viability

## Abstract

**Simple Summary:**

Our study identified specific conditions for optimal cell viability in enzymatic dissociation protocols across various endocrine tumors. For adrenal medullary tumors, the critical factor was the dissociation time, with 20 min of incubation proving most effective. In the case of adrenocortical tumors, using Collagenase IV or the MTDK enzyme kit, along with a post-dissociation procedure involving a debris removal kit, resulted in higher cell viability. No significant patterns were observed for thyroid carcinomas and pituitary neuroendocrine tumors, indicating that the effectiveness of dissociation protocols may vary significantly depending on the tissue type and its unique morphological characteristics. These findings underscore the importance of tailored dissociation protocols for different tumor types to ensure optimal outcomes in cell isolation processes.

**Abstract:**

The enzymatic dissociation of human solid tissues is a critical process for disaggregating extracellular matrix and the isolation of individual cells for various applications, including the immortalizing primary cells, creating novel cell lines, and performing flow cytometry and its specialized type, FACS, as well as conducting scRNA-seq studies. Tissue dissociation procedures should yield intact, highly viable single cells that preserve morphology and cell surface markers. However, endocrine tissues, such as adrenal gland tumors, thyroid carcinomas, and pituitary neuroendocrine tumors, present unique challenges due to their complex tissue organization and morphological features. Our study conducted a morphological examination of these tissues, highlighting the intricate structures and secondary degenerative changes that complicate the dissociation process. We investigated the effects of various dissociation parameters, including the types of enzymes, incubation duration, and post-dissociation purification procedures, such as debris removal and nontarget blood cell lysis, on the viability of cells derived from different tumor types. The findings emphasize the importance of optimizing tissue digestion protocols to preserve cell viability and integrity, ensuring reliable outcomes for downstream analyses.

## 1. Introduction

Isolating primary tumor cells is crucial for studying cell function in basic research [1] and for investigating tumor pathophysiology in clinical and regenerative medicine [2,3]. The primary goal of dissociation is to disaggregate and isolate viable single cells, avoiding cell clumping, aggregate formation, and the presence of debris or blood, thus ensuring the reliability of subsequent analyses [4].

The morphological features of endocrine tumor tissues present significant challenges for enzymatic dissociation, particularly due to their complex and sensitive nature. Endocrine tumors, including adrenal medullary and adrenocortical tumors, thyroid carcinomas, and pituitary neuroendocrine tumors (PitNETs), are characterized by intricate cellular architectures with specialized structures and dense extracellular matrix (ECM) networks rich in collagen and strong cell-cell adhesion [5,6]. Moreover, these tumors often exhibit secondary degenerative changes, including hemorrhages, necrosis, hyalinosis, fibrosis, calcification, and amyloid deposition that complicate the isolation process. The presence of multiple, closely packed cell types further complicates the enzymatic separation of individual cells without causing damage. Therefore, it is crucial to optimize dissociation conditions, including the types of enzymes, dissociation time, and post-dissociation purification procedures, tailored to each tissue type to ensure efficient cell isolation and preserve cell viability.

To digest the extracellular matrix of complex endocrine tumors, specific proteolytic enzymes are used, such as hyaluronidase, collagenases, dispase, trypsin, papain, and DNase or commercial enzyme cocktails. These enzymes dissociate the cell–cell contacts and the ECM-encompassing cells within the tumor tissue.

Collagenase types I and IV are crucial proteolytic enzymes extensively used for isolating primary cells from delicate tissues. They efficiently degrade collagen while preserving cell surface markers and viability [7,8]. Hyaluronidase degrades hyaluronic acid in the ECM, aiding in connective tissue dissociation and improving cell yield [9]. Trypsin is highly effective for cell dissociation with high viability, although some antigens are sensitive to its activity, potentially affecting downstream analyses [10]. Dispase targets fibronectin, collagen IV, and other ECM proteins, making it suitable for isolating epithelial cells in endocrine tumors [11]. Papain, a broad-spectrum proteolytic enzyme less destructive than other proteases, digests various ECM components, facilitating the release of viable single cells from diverse tissues [12]. DNase I prevents cell clumping by degrading DNA from lysed cells [13]. Selecting the appropriate enzymes is crucial for preserving cell integrity, viability, and surface markers during isolation.

Dissociation time is also a critical factor in tissue dissociation processes, significantly impacting cell viability and states. Overexposure to enzymes can damage cell integrity, functionality, and viability by activating stress signaling pathways, potentially introducing artifacts that distort the transcriptional profiles of individual cells [14]. While efforts have been made to mitigate these issues, they often remain limited to specific tissue types and protocols, leaving many artifacts unaddressed and necessitating the further refinement and validation of dissociation methods across diverse tissues.

Post-dissociation purification procedures such as debris removal (DRS) and red blood cell lysis (RBCS) are important for the quality and viability of isolated cells. Debris removal, based on density differences, allows viable cells to be separated from debris and dead cells [15]. For blood cell lysis, hypotonic buffers selectively lyse red blood cells (RBCs) by causing osmotic swelling and rupture, preserving the viability of other cells [16].

By using specific enzymes, precise incubation times, and post-dissociation procedures, enzymatic dissociation facilitates the efficient separation of cells while preserving their viability and molecular characteristics. This study assessed the impact of various enzymes, including Collagenase I, Collagenase IV, and commercial enzyme kits from Miltenyi Biotec, such as Multi Tissue Dissociation Kit (MTDK) and Neural Tissue Dissociation Kit (NTDK). Additionally, we examined the effects of post-dissociation purification steps, such as debris removal and nontarget blood cell lysis, on the viability of tumor cells. Other factors that could influence cell viability, such as centrifugation, filtration, temperature conditions, pipetting frequency, and chemical reagents, were consistent across all samples, except for the number of samples per tissue type, which varied slightly and may have had a minimal effect. Optimal dissociation parameters were identified for each type of endocrine tumor tissue sample, enhancing cell viability and maintaining cellular integrity for subsequent analyses.

## 2. Materials and Methods

### 2.1. Tissue Sampling

**Figure 1:**
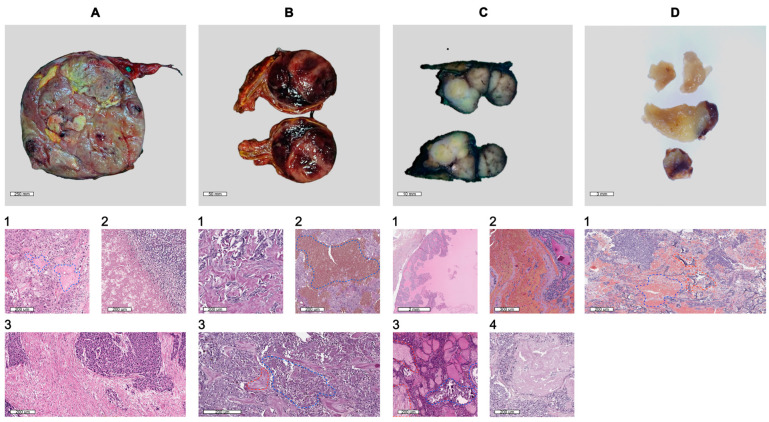
Images of endocrine tumor samples. (**A**) Gross image of the adrenal medullary tumor. 1. A microscopic image of the adrenal medullary tumor with hyalinized stroma; H&E; ×180. 2. A microscopic image of the adrenal medullary tumor with hemorrhage (blue dashed line); H&E; ×150. 3. A microscopic image of the adrenal medullary tumor hyalinized stroma (red dashed line) and necrosis (blue dashed line). In the areas of tumor necrosis, there is a large number of segmented neutrophils; H&E; ×150. (**B**) Gross image of the adrenocortical tumor. 1. A microscopic image of the adrenocortical tumor with hyalinized stroma (blue dashed line); H&E; ×150. 2. A microscopic image of the adrenocortical tumor with a large area of tumor necrosis. There is pronounced inflammatory infiltration around the necrotic area; H&E; ×150. 3. A microscopic image of the adrenocortical tumor with high fibrosis; H&E; ×180. (**C**) Gross image of the thyroid carcinoma. 1. A microscopic image of the nodular thyroid hyperplasia. Proliferation of thyrocytes with the formation of papillary structures and accumulation of colloid in follicles; H&E; ×80. 2. A microscopic image of the thyroid tissue with hemorrhage; H&E; ×150. 3. A microscopic image of the papillary thyroid carcinoma with hyalinized stroma (red dashed line) and calcification (blue dashed line); H&E; ×150. 4. A microscopic image of the medullary thyroid carcinoma with the deposition of homogeneous eosinophilic masses (amyloid); H&E; ×180. (**D**) Gross image of the PitNET. 1. A microscopic image of the PitNET with hemorrhage (blue dashed line) and bone fragments (red dashed line); H&E; ×150.

A total of 51 human adrenal gland neoplasms (including 25 adrenocortical tumors and 26 adrenal medullary tumors), 9 thyroid carcinomas, and 33 pituitary neuroendocrine tumors (PitNETs) were obtained from the Endocrinology Research Centre in Moscow, Russia (Figure 1). After sampling, the tissues were stored in a cold tissue storage solution (Miltenyi Biotec) until dissociation. All patients had their tumor specimens definitively diagnosed through imaging, surgery, and histopathological examination. Following the Declaration of Helsinki, the institution’s ethics and research committee approved the research protocol (Approval No. 16, dated 14.10.2020). All the study participants provided written informed consent.

### 2.2. Histopathological Examination

Tumor tissue samples were fixed in 10% buffered formalin (Leica Biosystems, Nussloch, Germany), processed in the histological staining system of Leica ASP6025, and embedded in paraffin (Leica Biosystems, Nussloch, Germany). Subsequently, paraffin sections with a thickness of 3 µm were cut from the paraffin-embedded tumor tissue samples using a microtome (Thermo Fisher HM 355 S, Thermo Fisher Scientific, Waltham, MA, USA) and were applied to slides treated with poly (l-lysine). The slides were then stained with hematoxylin (Leica Biosystems, Nussloch, Germany) and eosin (Leica Biosystems, Nussloch, Germany) following the standard procedure. All histological slides were scanned using a Leica Aperio AT2 system (Leica Biosystems, Nussloch, Germany) at 20× magnification for further analysis.

### 2.3. Enzymatic Dissociation

Approximately 200–220 mg of fresh adrenal gland neoplasm, thyroid carcinoma, or 7–10 mg of PitNET samples were washed in HBSS (Gibco, Thermo Fisher Scientific, Waltham, MA, USA); then, they were thoroughly minced on ice. The samples were then placed in a dissociating solution at 37 °C with gentle pipetting every 5 min.

The adrenal gland neoplasm samples were dissociated using 25 uL of either enzyme D Multi Tissue Dissociation Kit (MTDK) (Miltenyi Biotec, Bergisch Gladbach, Germany) or enzyme A Neural Tissue Dissociation Kit P (NTDK) (Miltenyi Biotec, Bergisch Gladbach, Germany) or 2 mg/mL of Collagenase IV (Gibco, Thermo Fisher Scientific, Waltham, MA, USA) or Collagenase I (Gibco, Thermo Fisher Scientific, Waltham, MA, USA) in a solution of 870 mM HBSS (Capricorn scientific, Ebsdorfergrund, Germany), 10% FBS (Gibco, Thermo Fisher Scientific, Waltham, MA, USA), and 20 mM HEPES (Gibco, Thermo Fisher Scientific, Waltham, MA, USA) for 20–35 min.

PitNET samples were dissociated with 10 uL of enzyme D MTDK (Miltenyi Biotec, Bergisch Gladbach, Germany) or 2 mg/mL Collagenase IV (Gibco, Thermo Fisher Scientific, Waltham, MA, USA) in a solution of 870 mM HBSS (Capricorn scientific, Ebsdorfergrund, Germany), 10% FBS (Gibco, Thermo Fisher Scientific, Waltham, MA, USA), and 20 mM HEPES (Gibco, Thermo Fisher Scientific, Waltham, MA, USA) for 7–15 min.

The thyroid carcinoma samples were dissociated using 35 uL of enzyme D MTDK (Miltenyi Biotec, Bergisch Gladbach, Germany) in a solution of 870 mM HBSS (Capricorn scientific, Ebsdorfergrund, Germany), 10% FBS (Gibco, Thermo Fisher Scientific, Waltham, MA, USA), and 20 mM HEPES (Gibco, Thermo Fisher Scientific, Waltham, MA, USA) for 20–30 min.

The homogenate was filtered in 3–5 mL of Wash Buffer (1× DPBS (Sigma-aldrich, Burlington, MA, USA), containing 10% FBS (Gibco, Thermo Fisher Scientific, Waltham, MA, USA), 20 mM HEPES (Gibco, Thermo Fisher Scientific, Waltham, MA, USA), and 6 mM glucose (Millipore Sigma, Burlington, MA, USA)) through a prewetted 70 μm cell culture filter (Miltenyi Biotec, Bergisch Gladbach, Germany) and then centrifuged for 5 min at 300× *g* (4 °C).

For samples with high blood and debris content, the red blood cells were lysed using Red Blood Cell Lysis Solution (RBCS, Miltenyi Biotec, Bergisch Gladbach, Germany), and dead cells were removed with the Dead Cell Removal Kit (DRS, Miltenyi Biotec, Bergisch Gladbach, Germany). The cells were then counted and assessed for viability using trypan blue staining on the Countess 3 (Thermo Scientific, Waltham, MA, USA). Lastly, the pellet was resuspended in a Wash Buffer volume of 100–400 uL, depending on the pellet size.

### 2.4. Statistical Data Analysis

Comparisons of cell viability between dissociation time points were performed using Welch’s one-way ANOVA (oneway.test function in R), followed by pairwise Welch’s *t*-tests (t.test function in R). Pairwise Wilcoxon rank-sum tests (wilcox.test function in R) were used to assess the differences in cell viability between samples grouped by enzyme type, RBCS, or DRS. For the line graphs, the data are presented as mean ± SD. All reported *p*-values were corrected for multiple testing using the Holm–Bonferroni method. Statistical significance is shown in the figures: ****: *p* ≤ 0.0001; ***: *p* ≤ 0.001; **: *p* ≤ 0.01; *: *p* ≤ 0.05; ns—not significant—*p* > 0.05.

## 3. Results and Discussion

### 3.1. Influence of Morphological Features on the Dissociation Process and Cell Viability

The process of dissociating and isolating cells from tumor tissues is complicated by various morphological features and secondary degenerative changes. Endocrine tumors often exhibit dense extracellular matrices and strong cell–cell adhesions, which hinder effective enzymatic dissociation. Additionally, the following aspects can further exacerbate the challenges in tissue dissociation: secondary degenerative changes, such as hemorrhages, particularly in adrenal medullary tumors (Figure 1(A2)); PitNETs (Figure 1(D1)); thyroid carcinomas (Figure 1(C2)); necrosis and fibrosis (commonly found in various tissues (Figure 1(B2,B3,A3))); hyalinosis, notably in adrenal gland tumors (Figure 1(A1,A3)); the calcification and accumulation of colloid, commonly seen in thyroid tumors (Figure 1(C1,C3)), etc. These changes are frequently observed in tumors of various localizations and arise from multiple factors. For instance, rapid tumor growth can lead to the formation of inadequate blood vessels, resulting in insufficient oxygenation and subsequent tissue hypoxia. This hypoxia particularly affects the central regions of tumors, causing necrosis due to limited blood supply [17]. Hyalinosis, associated with prolonged hypoxia and inflammation, causes collagen fibers to merge into dense, homogenous masses, complicating tissue digestion [18].

Specifically, dissociating PitNETs tissues is challenging due to the gland’s location in the sella turcica, a cavity in the sphenoid bone. The surgical removal of pituitary tumors can involve adjacent bone structures, leading to the presence of bone fragments in postoperative samples. These factors, combined with hemorrhage, which often results from the tumor’s inherent vascularity and the fragile nature of the blood vessels within it, complicate the isolation of viable cells for analysis [19].

The enzymatic dissociation of adrenocortical tumors is challenging due to the presence of a hyalinized stroma as well as in adrenal medullary tumors (Figure 1(B1,A1,A3)), fibrosis (Figure 1(B3)) and high lipid content. The dense, fibrous extracellular matrix enriched with hyaline restricts enzyme penetration, impeding the breakdown necessary for isolating individual cells [20]. Furthermore, the high lipid content creates a physical barrier and sequesters enzymes, reducing their effectiveness and complicating the purification process by mixing with cellular debris.

In thyroid carcinomas, homogeneous eosinophilic amyloid deposits often arise due to excessive calcitonin production by parafollicular C cells (Figure 1(C4)). These deposits form insoluble fibrillar structures that accumulate in the intercellular space [21]. The presence of amyloid deposits, fibrous tissue, and psammoma bodies (calcifications)—calcified concretions with a glassy, laminated appearance—creates substantial challenges during enzymatic dissociation [22] (Figure 1(C3,C4)). The dense, fibrous stroma and insoluble amyloid masses impede enzyme penetration, while the calcified psammoma bodies further complicate the dissociation process, thereby reducing the efficiency of isolating viable cells.

These factors collectively hinder efficient tissue dissociation and lower the yield of viable cells. Considering these morphological features and degenerative changes is essential for effective enzymatic dissociation. The accurate assessment and appropriate adjustment of the dissociation protocol, based on the tumor’s specific characteristics, are necessary to improve enzyme penetration, enhance the breakdown of structural barriers, and optimize the yield of viable cells. This involves selecting suitable enzymes, adjusting dissociation time, and implementing effective post-dissociation purification procedures for each tissue type.

### 3.2. Influence of Enzymatic Activity on Cell Viability

**Figure 2:**
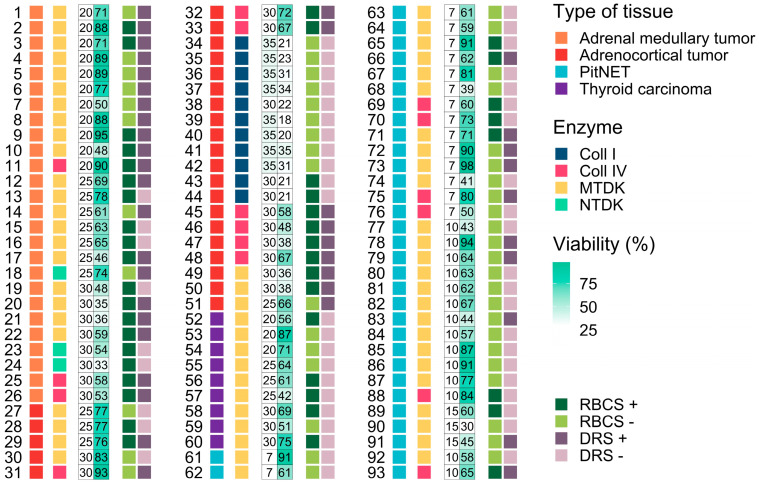
Representation of cell suspensions (n = 93) from four studied tumor tissue types, including adrenal medullary and adrenocortical tumors, thyroid carcinomas, and PitNETs (pituitary neuroendocrine tumors). The figure sequentially indicates the type of enzyme used, including Coll I (Collagenase I), Coll IV (Collagenase IV), MTDK (Multi Tissue Dissociation Kit), and NTDK (Neural Tissue Dissociation Kit), along with the dissociation time (min), cell viability (%), and post-dissociation procedures. The post-dissociation procedures include RBCS +/− (Red Blood Cell Lysis Solution) and DRS +/− (Dead Cell Removal Solution).

We assessed the impact of different enzymatic treatments on cell viability across various endocrine tumor samples, including adrenal medullary (n = 26), adrenocortical (n = 25), thyroid carcinoma (n = 9), and PitNETs (n = 33) (Figure 2). Our study utilized enzymes, such as Collagenase I and IV, as well as commercial enzyme kits MTDK and NTDK from Miltenyi Biotec. We used the trypan blue staining assay to measure cell viability.

**Figure 3:**
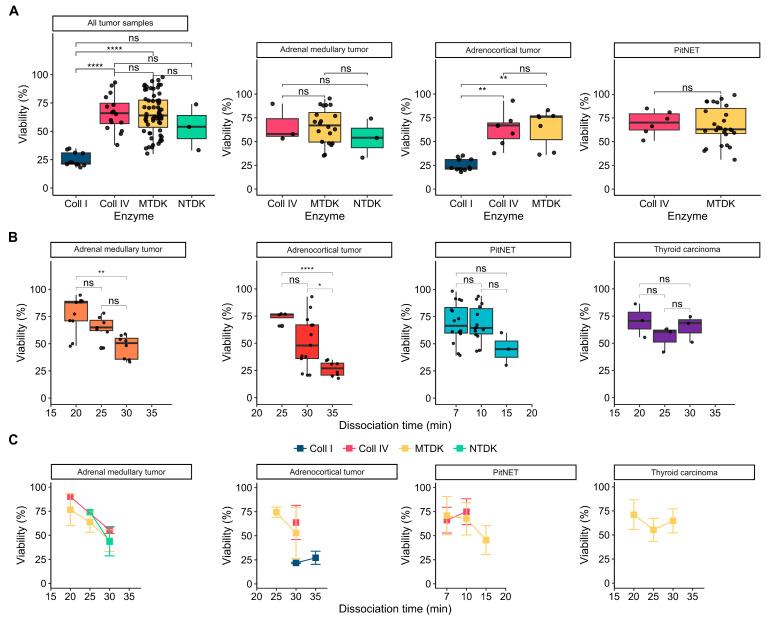
Comparison of the effects of various enzyme types and dissociation time intervals on the viability of cell suspensions (n = 93) from four studied tumor tissue types. (**A**) Box plots compare the influence of enzyme types, including Coll I (Collagenase I), Coll IV (Collagenase IV), MTDK (Multi Tissue Dissociation Kit), and NTDK (Neural Tissue Dissociation Kit), on cell viability across different tissue types: adrenal medullary tumor (n = 26), adrenocortical tumor (n = 25), PitNET (n = 33) samples, and all tumor samples combined (n = 84). (**B**) Box plots compare the influence of dissociation time intervals (min) on cell viability across tissue types: adrenal medullary tumor (n = 26), adrenocortical tumor (n = 25), PitNET (n = 33), and thyroid carcinoma (n = 9) samples. (**C**) The line graphs compare cell viability over dissociation time intervals (min) based on enzyme types for the adrenal medullary tumor (n = 26), adrenocortical tumor (n = 25), PitNET (n = 33), and thyroid carcinoma (n = 9) samples. Comparisons of cell viability between dissociation time intervals were conducted using Welch’s one-way ANOVA (oneway.test function in R), followed by pairwise Welch’s *t*-tests (t.test function in R). Pairwise Wilcoxon rank-sum tests (wilcox.test function in R) were used to evaluate differences in cell viability between samples grouped by enzyme type, RBCS, or DRS. The data in the line graphs are presented as mean ± SD. The statistical significance is indicated as follows: ****: *p* ≤ 0.0001; **: *p* ≤ 0.01; *: *p* ≤ 0.05; ns (not significant): *p* > 0.05.

Specifically, MTDK and NTDK kits, as well as Collagenase IV, were tested on adrenal medullary tumors. Collagenases IV and I, along with the MTDK kit, were tested on adrenocortical tumors. The MTDK kit was used on thyroid carcinomas, and MTDK and Collagenase IV were tested on PitNETs (Figure 2 and Figure 3A). Our findings indicate significant differences in cell viability for adrenocortical tumor samples treated with Collagenase I and Collagenase IV, with means of 25.18 ± 6.23% and 66 ± 15.51%, respectively (*p* = 0.0039, pairwise Wilcoxon rank-sum test) (Figure 3A; Tables S1 and S2 in the Supplementary File). A similar significant difference in viability was observed between Collagenase I and MTDK, with mean viabilities of 25.18 ± 6.23% and 65.75 ± 17.84%, respectively (Figure 3A; Tables S1 and S2 in the Supplementary File).

Across all tumor types, there was a similar range of cell viability when comparing Collagenase I and Collagenase IV, with mean viabilities of 25.18 ± 6.23% and 66 ± 15.51%, respectively, demonstrating highly significant differences (*p* = 0.00007; pairwise Wilcoxon rank-sum test). Additionally, strong significant differences were noted between Collagenase I and MTDK enzymes, with means of 25.18 ± 6.23% and 65.75 ± 17.84%, (*p* = 0.000001, pairwise Wilcoxon rank-sum test) (Figure 3A; Tables S1 and S2 in the Supplementary File). Among the enzymes tested, Collagenase I resulted in the lowest cell survival rates across all tissue samples, while Collagenase IV was associated with the highest survival rates, indicating a superior preservation of cell viability.

### 3.3. Influence Dissociation Time on Cell Viability

We further investigated the impact of dissociation time on cell viability by analyzing samples from adrenal medullary tumors, thyroid carcinomas, adrenocortical neoplasms, and pituitary neuroendocrine tumors (PitNETs). The evaluated incubation times were 20, 25, and 30 min for adrenal medullary tumors and thyroid carcinomas, 25 and 30 min for adrenocortical neoplasms, and 7, 10, and 15 min for PitNETs. These intervals were selected based on the unique morphological characteristics of each tissue type, the enzyme type and concentration, and the sensitivity of the cells.

Significant differences in cell viability were observed in adrenal medullary tumor samples when comparing the 20 and 30 min dissociation times (*p* = 0.0013, pairwise Student’s *t*-test). Specifically, mean viability decreased from 77.82 ± 16.3% to 47 ± 10.77% (Figure 3B; Tables S1 and S2 in Supplementary File). Similarly, significant differences in cell viability were observed in adrenocortical tumor samples when comparing the 25 and 35-min (*p* = 0.00001; pairwise Student’s *t*-test) and the 30 and 35 min (*p* = 0.037; pairwise Student’s *t*-test) dissociation times. Mean viability decreased from 74 ± 5.35% to 26.62 ± 6.82% and from 51.08 ± 24.16% to 26.62 ± 6.82%, respectively.

These results indicate that extending the dissociation time to 35 min may compromise cell viability, reducing it by less than 30%. Conversely, no significant correlation between dissociation time and cell viability was noted for PitNET and thyroid carcinoma samples (Figure 3B,C). This suggests that prolonged dissociation may negatively impact cell viability in some tumor types while having negligible effects in others, highlighting the necessity for optimizing dissociation conditions specific to each tissue type.

### 3.4. Influence of Additional Post-Dissociation Purification Procedures on Cell Viability

**Figure 4:**
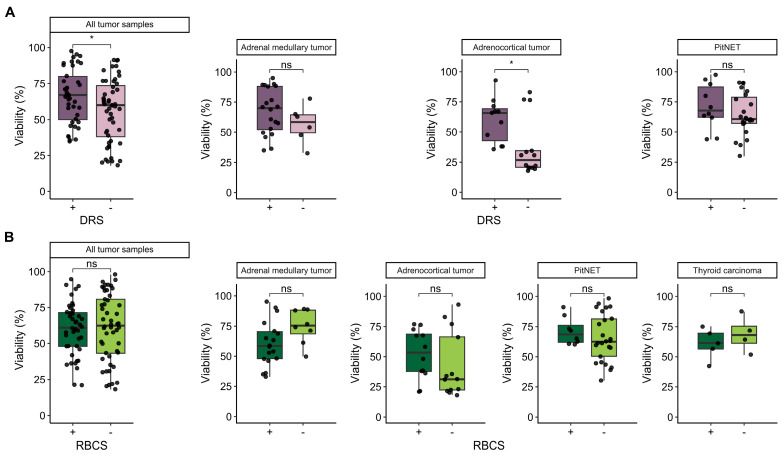
Comparison of the effects of the post-dissociation procedures: DRS (Dead Cell Removal Solution) and RBCS (Red Blood Cell Lysis Solution) on the viability of cell suspensions from studied tumor tissue types. (**A**) Box plots compare the influence of DRS +/− on cell viability across adrenal medullary tumor (n = 26), adrenocortical tumor (n = 25), PitNET (n = 33) samples, and all tumor samples combined (n = 84). (**B**) Box plots compare the influence of RBCS +/− on cell viability across adrenal medullary tumor (n = 26), adrenocortical tumor (n = 25), PitNET (n = 33) samples, thyroid carcinoma (n = 9) samples, and all tumor samples combined (n = 93). Comparisons of cell viability between dissociation time intervals were conducted using Welch’s one-way ANOVA (oneway.test function in R), followed by pairwise Welch’s *t*-tests (t.test function in R). Pairwise Wilcoxon rank-sum tests (wilcox.test function in R) were used to evaluate the differences in cell viability between samples grouped by RBCS or DRS. Statistical significance is indicated as follows: *: *p* ≤ 0.05, ns (not significant): *p* > 0.05.

In order to optimize the preparation of isolated cells for subsequent analyses, we assessed the impact of additional post-dissociation purification procedures, such as DRS and RBCS, focusing on the elimination of nontarget and dead cells to preserve viable ones (Figure 4). Our study found significant differences in cell viability between samples processed with and without DRS (*p* = 0.03; Wilcoxon rank-sum test), with mean viabilities of 66.32 ± 18.78% and 56.1 ± 21.76%, respectively (Figure 4A; Tables S1 and S2 in Supplementary File). Notably, DRS significantly improved cell viability, especially in adrenocortical tumor cells, where viabilities increased from 36.71 ± 23.6% to 59.91 ± 18.21% (*p* = 0.032; Wilcoxon rank-sum test) (Figure 4A; Tables S1 and S2 in the Supplementary File). This improvement is attributed to the reduction in mechanical stress on viable cells, the minimization of potential contaminants, and the overall provision of a cleaner sample. During the dissociation of thyroid carcinoma, no debris was observed, so a post-dissociation procedure using DRS to remove debris was deemed unnecessary.

In contrast, the examination of red blood cell removal revealed no significant differences in cell viability between samples treated with and without RBCS (Figure 4B). This indicates that the RBCS procedure does not adversely affect the viability of target cells. This finding aligns with the understanding that osmotic lysis selectively targets RBCs due to the presence of the abundant membrane Cl^−^/HCO_3_^−^ anion exchanger known as Band 3, which is unique to mature RBCs [23]. Consequently, the removal of RBCs does not compromise the integrity of other cell types in the sample.

## 4. Conclusions

Tissue dissociation is crucial for various areas of cell biology research. However, in many cases, standardized dissociation methods for different tissue samples are lacking. In our study, we focused on endocrine gland tumors, but there are numerous other human and animal tissues with similarly complex morphological structures that can impact tissue dissociation. When examining enzymes, we found that Collagenase IV and MTDK are the most effective for dissociation while maintaining viability. These enzymes are suitable for tissues with necrosis, permanent scar tissue, high lipid content, and increased hyaline content. Despite the dense nature of these tissues, the timing of dissociation is crucial, as prolonged dissociation can lead to loss of viability and cell death. The addition of RBCS is essential, as it does not affect cell suspensions but significantly clears nontarget blood cells from the suspension, making further work much easier. DRS should be used with all tissue types as it improves cell survival and removes extrinsic noise from debris or dead cells. It is important to note that our study has several limitations. One critical issue is the challenge of selecting and accounting for all dissociation conditions to obtain a sufficient number of viable cells, as tissues are often not uniform like cell lines. Other challenges include the limited number of high-quality living cells in the original tissue, the inadequate representation of various enzyme types in the laboratory, and difficulties in the quality, normalization, and standardization of data. Despite these limitations, cell survival studies are crucial, as cell suspensions are utilized in a wide range of modern research, including the popular and cutting-edge method of studying the transcriptome of single cells. Our research aims to streamline the production of whole, living single cells not only from endocrine tissues but also from various other human and animal tissues.

## Data Availability

All data generated or analyzed during this study are included in this published article.

## Supplementary Materials

The following supporting information can be downloaded at https://www.mdpi.com/article/10.3390/biology13090665/s1, Table S1: Cell viability mean ± SD for all sample groups and dissociation parameters; Table S2: Statistical data analysis of sample groups with test names and *p*-values.

## References

[1] Zhang S., Kuhn J.R. (2013). Cell Isolation and Culture.

[2] Singh S.K., Hawkins C., Clarke I.D., Squire J.A., Bayani J., Hide T., Henkelman R.M., Cusimano M.D., Dirks P.B. (2004). Identification of Human Brain Tumour Initiating Cells. Nature.

[3] Carvalho P.P., Gimble J.M., Dias I.R., Gomes M.E., Reis R.L. (2013). Xenofree Enzymatic Products for the Isolation of Human Adipose-Derived Stromal/Stem Cells. Tissue Eng. Part C Methods.

[4] Reichard A., Asosingh K. (2019). Best Practices for Preparing a Single Cell Suspension from Solid Tissues for Flow Cytometry. Cytometry. Part A J. Int. Soc. Anal. Cytol..

[5] Rozario T., DeSimone D.W. (2010). The Extracellular Matrix in Development and Morphogenesis: A Dynamic View. Dev. Biol..

[6] Gumbiner B.M. (1996). Cell Adhesion: The Molecular Basis of Tissue Architecture and Morphogenesis. Cell.

[7] Tanaka K., Okitsu T., Teramura N., Iijima K., Hayashida O., Teramae H., Hattori S. (2020). Recombinant Collagenase from Grimontia Hollisae as a Tissue Dissociation Enzyme for Isolating Primary Cells. Sci. Rep..

[8] Shi L., Ermis R., Garcia A., Telgenhoff D., Aust D. (2010). Degradation of Human Collagen Isoforms by Clostridium Collagenase and the Effects of Degradation Products on Cell Migration. Int. Wound J..

[9] Balashova A., Pershin V., Zaborskaya O., Tkachenko N., Mironov A., Guryev E., Kurbatov L., Gainullin M., Mukhina I. (2019). Enzymatic Digestion of Hyaluronan-Based Brain Extracellular Matrix in Vivo Can Induce Seizures in Neonatal Mice. Front. Neurosci..

[10] Corver W.E., Cornelisse C.J., Hermans J., Fleuren G.J. (1995). Limited Loss of Nine Tumor-Associated Surface Antigenic Determinants after Tryptic Cell Dissociation. Cytometry.

[11] Volovitz I., Shapira N., Ezer H., Gafni A., Lustgarten M., Alter T., Ben-Horin I., Barzilai O., Shahar T., Kanner A. (2016). A Non-Aggressive, Highly Efficient, Enzymatic Method for Dissociation of Human Brain-Tumors and Brain-Tissues to Viable Single-Cells. BMC Neurosci..

[12] Hussain R.Z., Miller-Little W.A., Doelger R., Cutter G.R., Loof N., Cravens P.D., Stüve O. (2018). Defining Standard Enzymatic Dissociation Methods for Individual Brains and Spinal Cords in EAE. Neurol. Neuroimmunol. Neuroinflamm..

[13] Jager L.D., Canda C.-M.A., Hall C.A., Heilingoetter C.L., Huynh J., Kwok S.S., Kwon J.H., Richie J.R., Jensen M.B. (2016). Effect of Enzymatic and Mechanical Methods of Dissociation on Neural Progenitor Cells Derived from Induced Pluripotent Stem Cells. Adv. Med. Sci..

[14] Van den Brink S.C., Sage F., Vértesy Á., Spanjaard B., Peterson-Maduro J., Baron C.S., Robin C., van Oudenaarden A. (2017). Single-Cell Sequencing Reveals Dissociation-Induced Gene Expression in Tissue Subpopulations. Nat. Methods.

[15] Miller R.G., Phillips R.A. (1969). Separation of Cells by Velocity Sedimentation. J. Cell. Physiol..

[16] Singh S., Ponnappan N., Verma A., Mittal A. (2019). Osmotic Tolerance of Avian Erythrocytes to Complete Hemolysis in Solute Free Water. Sci. Rep..

[17] Baloch Z.W., Asa S.L., Barletta J.A., Ghossein R.A., Juhlin C.C., Jung C.K., LiVolsi V.A., Papotti M.G., Sobrinho-Simões M., Tallini G. (2022). Overview of the 2022 WHO Classification of Thyroid Neoplasms. Endocr. Pathol..

[18] Mete O., Erickson L., Juhlin C., Krijger R., Sasano H., Volante M., Papotti M. (2022). Overview of the 2022 WHO Classification of Adrenal Cortical Tumors. Endocr. Pathol..

[19] Mortini P., Albano L., Barzaghi L.R., Losa M. (2021). Pituitary Surgery. La Presse Médicale.

[20] Sun B. (2021). The Mechanics of Fibrillar Collagen Extracellular Matrix. Cell Rep. Phys. Sci..

[21] Lebrun L., Salmon I. (2024). Pathology and New Insights in Thyroid Neoplasms in the 2022 WHO Classification. Curr. Opin. Oncol..

[22] Ferreira L., Gimba E., Vinagre J., Sobrinho-Simões M., Soares P. (2020). Molecular Aspects of Thyroid Calcification. Int. J. Mol. Sci..

[23] Chernyshev A.V., Tarasov P.A., Semianov K.A., Nekrasov V.M., Hoekstra A.G., Maltsev V.P. (2008). Erythrocyte Lysis in Isotonic Solution of Ammonium Chloride: Theoretical Modeling and Experimental Verification. J. Theor. Biol..

